# Enhanced recognition of disgusted expressions occurs in spite of attentional avoidance at encoding

**DOI:** 10.3389/fpsyg.2022.1063073

**Published:** 2023-01-04

**Authors:** Tom Zalmenson, Omer Azriel, Yair Bar-Haim

**Affiliations:** School of Psychological Sciences, Tel Aviv University, Tel Aviv, Israel

**Keywords:** emotion, facial expressions, disgust, attention, memory

## Abstract

**Introduction:**

Negative emotional content is prioritized in memory. Prioritized attention to negative stimuli has been suggested to mediate this valence-memory association. However, research suggests only a limited role for attention in this observed memory advantage. We tested the role of attention in memory for disgusted facial expressions, a powerful social–emotional stimulus.

**Methods:**

We measured attention using an incidental, free-viewing encoding task and memory using a surprise memory test for the viewed expressions.

**Results and Discussion:**

Replicating prior studies, we found increased attentional dwell-time for neutral over disgusted expressions at encoding. However, contrary to the attention-memory link hypothesis, disgusted faces were better remembered than neutral faces. Although dwell-time was found to partially mediate the association between valence and memory, this effect was much weaker than the opposite direct effect. These findings point to independence of memory for disgusted faces from attention during encoding.

## Introduction

Negatively valenced stimuli have advantage in memory (Hamann, 2001; LaBar and Cabeza, 2006). It has been suggested that attention may regulate memory stabilization (e.g., Irwin and Zelinsky, 2002; Kandel, 2007). Specifically, because negative stimuli are thought to draw greater attention than neutral stimuli (Öhman et al., 2001), this prioritized processing could translate into advantaged memory for negative information (Kensinger and Corkin, 2004). However, experimental research generally points to a limited role for attention in memory advantage for negative information (Talmi and McGarry, 2012; Talmi, 2013). Here, we tested attention-memory links in relation to a negative social cue - disgusted expressions.

Disgusted facial expressions are informative (Ekman, 1993), signal threat-related aspects of the social environment (Giner-Sorolla and Espinosa, 2011), and evoke strong neural responses, similar to those evoked by disgusting stimuli (Wicker et al., 2003). Clarifying the role of specific cognitive functions such as attention and memory in the processing of disgust facial expressions can contribute to the understanding of social information processing and to the way people interpret social events. For example, it has been suggested that biased attention and/or memory for disgust social stimuli could underlie the phenomenon of post-event processing, defined as repeated consideration and potential reconstruction of one’s performance following a social situation (Brozovich and Heimberg, 2008), which for some people occur immediately after experiencing social events (Rachman et al., 2000).

Attention research suggests a pattern of increased initial orienting followed by attentional avoidance of disgust stimuli (Carretié, 2014; Armstrong et al., 2019; for a review see Knowles et al., 2019). It has been suggested that the attentional initial orienting toward disgust stimuli reflects a general tendency to orient toward threat (Öhman et al., 2001), whereas the late avoidance of disgust stimuli could reflect an emotion regulation strategy aimed at reducing the displeasure of the exposure (Knowles et al., 2019). Studies employing disgusted facial expressions as stimuli in free-viewing eye-tracking tasks also indicate reduced dwell-time on disgusted relative to neutral or other negative facial expressions (e.g., Liu et al., 2015; Lazarov et al., 2016).

Hirsch et al. (2006) advanced a ‘combined biases hypothesis’, proposing that a cognitive bias in one stage of information processing could influence the processing in later stages. Thus, attentional avoidance of disgusted facial expressions, and therefore reduced procesing of faces from this category, could result in poorer memory for such expressions. However, experimental information regarding memory for disgusted vs. neutral facial expressions is scarce. One study found better memory for digust faces relative to neutral faces in an n-back task, in which participants view stimuli in a sequential manner and were requested to report whether the presented stimulus is identical to the stimulus presented n steps back (Román et al., 2015). This study suggests a preference in memory for disgust faces, but does not inform regarding the possible effect of attentional avoidance on memory. To our knowledge, no study to date explored the effect of reduced attention for disgusted facial expressions on later memory for the same faces.

Here, we test attention-memory associations in the context of disgusted vs. neutral facial expressions. Specifically, we asked whether attentional avoidance of disgusted expressions, measured using dwell-time on disgusted vs. neutral faces in a free-viewing task, would be associated with poorer recognition memory for disgust relative to neutral faces. We expected that: (a) based on prior findings (Liu et al., 2015; Lazarov et al., 2016; Armstrong et al., 2019), dwell-time will be shorter on disgusted relative to neutral facial expressions; and (b) better memory for neutral over disgusted expressions would emerge if indeed attention plays a central role in memory for the presented faces. In contrast, if attention plays a limited role in advantaged memory for disgusted expressions, as suggested in studies of non-face negative stimuli (Talmi and McGarry, 2012; Talmi, 2013), then a memory advantage for disgusted over neutral expressions should be expected regardless of the attentional dwell time patterns noted during encoding. Finally, we explored whether attentional dwell time during encoding mediated memory performance as a function of facial expression.

## Materials and methods

### Participants

Participants were 50 undergraduate students (29 females, mean age = 24.1 years, SD = 2.87, range = 20–36), who received course credit for their participation. The study was approved by the local Institutional Review Board.

### Measures

#### The free viewing attention task (encoding)

Gaze patterns were assessed using an established eye-tracking free-viewing task (Lazarov et al., 2016, 2021; Klawohn et al., 2020) adapted for the current study. The task was executed using the Experiment Builder software (version 2.1.140; SR Research Ltd., Mississauga, Ontario, Canada). Chromatic photographs of 16 male and 16 female models, each showing a disgusted and a neutral facial expression, were taken from the Karolinska Directed Emotional Faces database (KDEF; Lundqvist et al., 1998). To ensure that results were not specific to certain faces, the 64 images were randomly divided into 2 sets of 32 images each, in which each of the 32 models appeared in one facial expression only, i.e., if a model appeared with a disgusted facial expression in one set, they would appear with a neutral expression in the other set. Each set contained 16 disgusted and 16 neutral faces, with equal representation for both genders for each facial expression. Sets were counter-balanced between participants.

**Figure 1:**
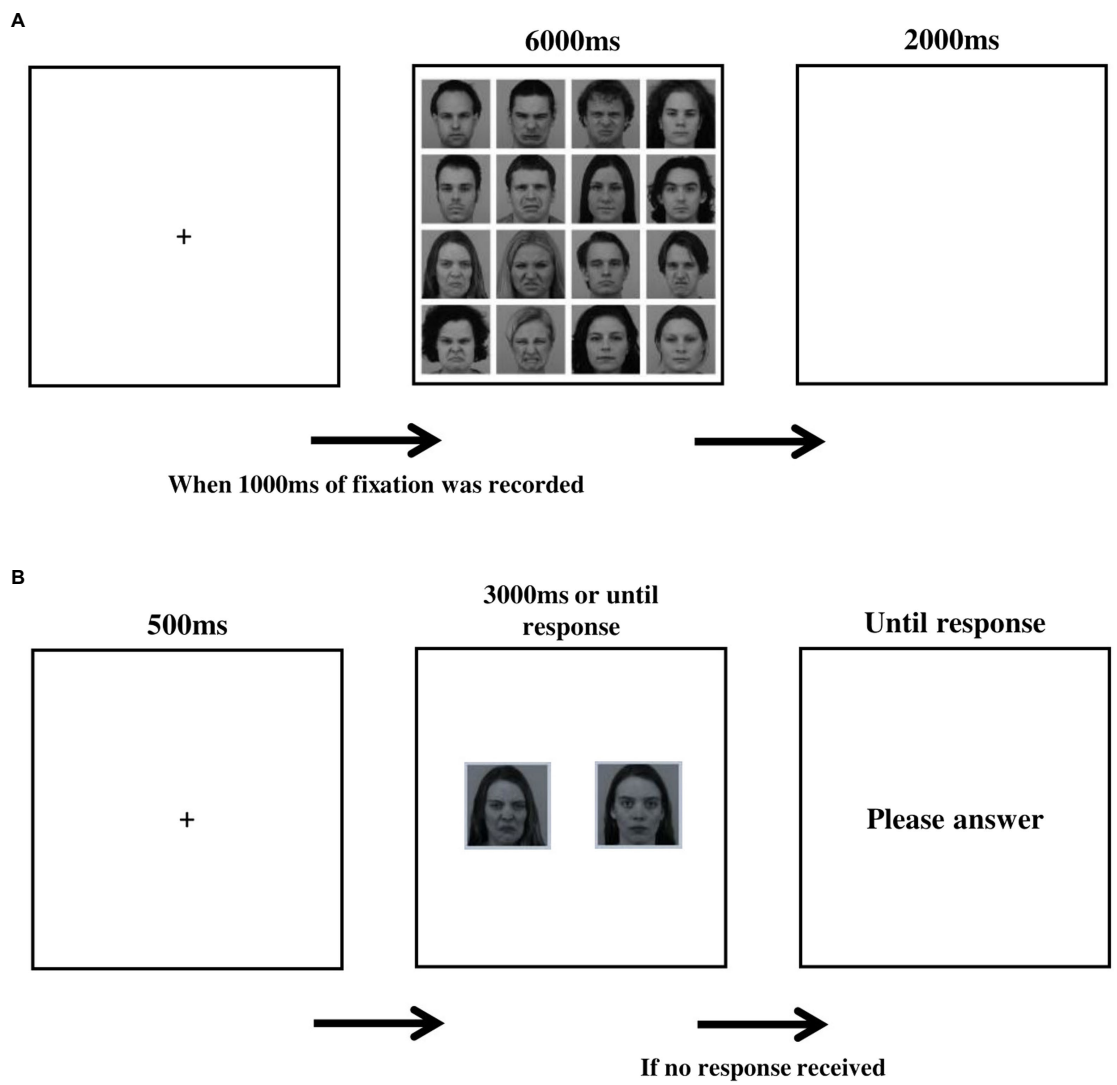
**(A)** Schematic illustration of a trial in the encoding stage. **(B)** Schematic illustration of a trial in the recognition test.

Stimuli were arranged in 4 × 4 matrices of 16 faces each (950 × 950 pixels). Half of the faces in each matrix were disgusted and half neutral. Each face extended 238 × 238 pixels, including an 11-pixel white margin on every edge. Each face appeared only once in any single matrix in a random position, ensuring each matrix contains 8 male and 8 female faces and that the 4 inner facial expressions contained 2 disgusted and 2 neutral faces. The set-up of a single trial is depicted in Figure 1A. Each trial began with a fixation-cross, shown until a fixation of 1,000 ms was recorded, followed by the presentation of a matrix (6,000 ms). The trial ended with a blank screen (2000 ms). Sixty different matrices were presented in two blocks of 30 matrices each, with a 1-min break between blocks. Each model appeared 15 times per block (i.e., 30 times with the same facial expression during encoding). Participants were instructed to look at the faces in a matrix in any way they liked until it disappeared.

Eye movements were recorded using a remote high-speed eye-tracker (Eye-Link 1,000 Plus, SR Research, Ltd., Ottawa, Ontario, Canada). Sampling rate was 1,000 Hz. Participants sat 70 cm from the monitor. The stimuli were presented on a 24 inch BenQXL2430 monitor (screen resolution of 1920 × 1080 pixels). Eye-tracking data was processed using Data Viewer software (SR Research, Ltd., Ottawa, Ontario, Canada). Two Areas of Interest (AOIs) were defined for each of the 60 matrices, the 8 disgust faces (i.e., the disgust AOI) and the 8 neutral faces (i.e., the neutral AOI). Fixations were defined as ≥100 ms fixation within 1-degree visual angle. Total dwell time per AOI (disgust/neutral) was calculated as the averaged total dwell time on each of the AOIs across the 60 matrices. Internal consistency for this measure in our sample was 0.79 for the disgust AOI and 0.92 for the neutral AOI.

#### Recognition memory test

All 32 models displayed in the encoding task appeared in the recognition memory test, along with 8 new model faces (4 males) selected from the same faces database (foil trials), for a total of 40 memory recognition trials. The average disgusted intensity scores in the two sets presented at encoding, and in the foil trials were taken from the validation data for the KDEF database (Goeleven et al., 2008) and did not differ between the two sets (M set A = 0.67; M set B = 0.61; M foil = 0.52; *F*(2, 37) = 0.93, *p* = 0.4). The set-up of a single trial in the memory test is depicted in Figure 1B. Each trial began with a fixation-cross (500 ms), after which pictures of neutral and disgusted facial expressions of the same model appeared side-by-side (3,000 ms). Face pairs were presented in a random order, and the location of the disgusted face within each pair was counterbalanced across trials. Before the memory recognition trials began, participants were told that each model to be presented appeared in the free viewing task in only one of the two facial expressions, and that based on their memory they are to determine which one it was. Responses were recorded *via* keyboard button press: ‘z’ (for the left image) and ‘/‘(for the right image). Foil trials were used to assess potential response bias (i.e., a general tendency to choose disgusted or neutral faces in the lack of actual memory for the foil faces). Three measures were derived from the memory recognition task: (a) memory accuracy scores, calculated for each facial expression (disgusted and neutral) as the percent of correct responses from the 16 relevant test trials; (b) memory bias, calculated by subtracting the percent of correct responses for neutral expressions from the percent of correct responses for disgusted expressions. A score above zero represents a memory bias in favor of disgusted faces; and (C) response bias scores, calculated as the percent of disgust responses made by the participant in the foil trials. A response bias score above 0.5 reflects a bias toward reporting that the disgusted expression was seen before.

#### Distractor task

To reduce the risk for recency effects on memory in the transition from encoding to recall (Broadbent and Broadbent, 1981), participants were administered a 4-min distractor task consisting of simple arithmetic operations (e.g., “258 + 14 =?”), immediately following the encoding phase.

### Procedure

Consenting participants were seated in front of the eye-tracking monitor located in a quiet room. A 5-point gaze calibration and validation was performed until visual deviation of below 0.5° on the X and Y axis was achieved. Participants then completed the free viewing task, followed by a 5-min retention interval (a break for 1 min and the distractor task for 4 min), and then performed the recognition memory test. Finally, participants were debriefed and dismissed.

#### Data analysis

To examine differences in attention to disgusted vs. neutral facial expressions, we conducted a one-way repeated-measures ANOVA on the mean averaged total dwell time on disgust/neutral AOIs, with AOI (disgust, neutral) as a within subject factor. To examine differences in memory for disgusted and neutral expressions, a one-way ANOVA was conducted with accuracy as the dependent factor and facial expression (disgust, neutral) as a within-subject factor.

Item-level mediation analysis was computed to describe the three-way associations between emotional valance, attention, and memory. For each participant, we calculated the total dwell-time per specific face image, and an average dwell-time score for all images. We then subtracted the former from the latter to create a dwell-time bias score for each image viewed by the participant. We then averaged this dwell-time bias score for each image over all participants, to create an average dwell-time bias score per image, reflecting attention allocation toward this particular face during encoding. In addition, we calculated an accuracy score for each image, based on how many participants correctly recognized its valance in the memory test, reflecting its retention in memory. Finally, valance was scored for each image as a binary variable with 1 for disgust, and 0 for neutral. The attention-bias, accuracy, and valance scores for each image were entered into a mediation model (Hayes, 2017), with attention-bias as the mediator, using the PROCESS macro v.3.5.3 software (model 4) for SPSS (SPSS, Chicago). Bias-corrected bootstrap confidence intervals were used for inference about indirect effects, with 95% confidence interval and 10,000 bootstrap samples. The mediator variables are considered significant if the confidence interval does not include zero.

Finally, a one-sample t-test was conducted to examine the deviation of a mean response bias from 0.5. An alpha level of <0.05 was used for all analyzes.

#### Power analysis

Power analysis for sample size estimation used GPower 3.1 (Faul et al., 2009). Based on data from Lazarov et al. (2021) of 20 healthy participants, the effect size for preference of neutral over threat faces was ($\eta_{p}^{2}$) = 0.39. With an alpha = 0.05 and power = 0.95 the projected sample size needed to detect a significant effect was *N* = 23. Based on data from Talmi et al. (2007) who studied 48 participants, the reported memory advantage for negative over neutral pictures is ($\eta_{p}^{2}$) = 0.75. This very large effect size indicates an even smaller required sample size than that indicated by the attention effect. The effect size of the association between attention and memory for disgusted faces is unknown. Therefore, to ascertain statistical power for the two separate effects of attention and memory we opted for a sample size of *N* = 50.

## Results

### Attention

**Figure 2:**
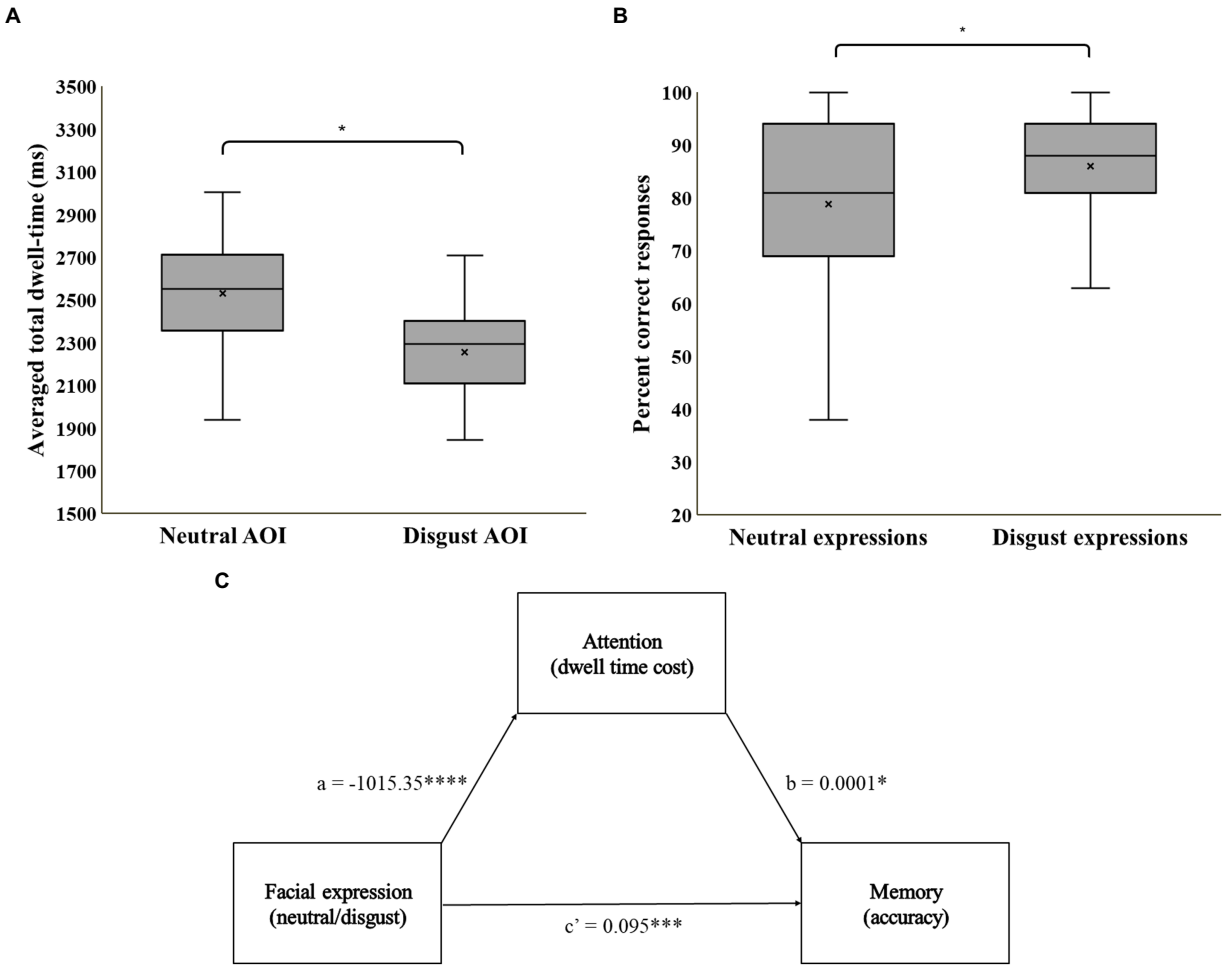
**(A)** Mean averaged total dwell time by AOI; **(B)** Percentage of correct recognition by expression. **(C)** A statistical diagram of the simple mediation model. * = *p* < 0.05, *** = *p* < 0.001, **** = *p* < 0.0001.

A repeated-measures ANOVA on total mean dwell time, with AOI as a within-subject variable, and set-type as a between-subjects variable revealed no main effect for set-type, or an AOI-by-set-type interaction (*p*s > 0.18). We therefore collapsed across set-type in all further analyzes. Mean dwell times in milliseconds by AOI are displayed in Figure 2A. Replicating the results of previous studies applying the free-viewing attention task, participants in the current study dwelled longer on neutral faces (*M* = 2,531, SD = 392) compared to disgust faces (*M* = 2,257, SD = 348), *F*(1,49) = 10.2, *p* = 0.002, $\eta_{p}^{2}$=0.17.

### Memory

A repeated measures ANOVA on percent of correct responses, with expression (disgust, neutral) as a within-subject variable and set-type (A, B) as a between-subjects variable, revealed no main effect for set-type or for expression-by-set-type interaction (*p*s > 0.05). We therefore collapsed across set-type in all further analyzes. Percent of correct responses by expression are displayed in Figure 2B. Participants displayed better memory for disgust (*M* = 86%, SD = 12) than for neutral (*M* = 79%, SD = 17) expressions, *F*(1,49) = 5.5, *p* = 0.02, $\eta_{p}^{2}$=0.10. No significant deviation from chance performance (0.5) was noted for mean response bias score in the foil trials (*M* = 0.53, SD = 0.24), *t*(49) = 0.81, *p* = 0.42, ruling out a general response bias.

### Mediation of the valence-memory association by attention

Mediation model coefficients are summarized in Figure 2C. Results indicate a direct effect of facial expression on memory, *b* = 0.095, SE = 0.02, *p* < 0.001, 95% CI (0.73, 0.80), an effect of facial expression on attention, *b* = −1015.35, SE = 250.007, *p* < 0.001, 95% CI (−1515.11, −515.59), and an effect of attention on memory, *b* = 0.00001, SE = 0.00001, *p* < 0.05, 95% CI (0.00001, 0.0001). Importantly, the bias corrected bootstrap confidence interval for the indirect effect does not include zero, indicating a significant inconsistent mediation effect of attention on the association between facial expression and memory, Effect = −0.03, BootSE = 0.015, 95% CI (−0.06, −0.004). This pattern of results suggests that although greater dwell time on any specific face during encoding is indeed associated with better memory of this face, the effect of enhanced memory for threat faces prevails despite the lower dwell time on disgust faces relative to neutral faces. Indeed, the direct effect of valence on memory when accounting for the indirect path (c’) is larger than the total effect of valence on memory, Effect = 0.065, SE = 0.025, *p* < 0.05, 95% CI [0.015, 0.115].

### Discussion

Replicating the results of prior studies applying the free-viewing attention task in non-clinical populations (e.g., Liu et al., 2015; Lazarov et al., 2016, 2021), participants in the current study dwelled longer on neutral relative to disgusted faces. Despite this potential attentional advantage for neutral faces during encoding, participants displayed a strong recognition memory advantage for disgust faces, a finding that corresponds with a previously shown memory advantage for disgusting content (e.g., Chapman et al., 2013; Moeck et al., 2021). The mediation analysis indicates a small indirect effect of valence on memory through attention allocation, which here was not strong enough to overcome the much stronger positive direct effect of valence on memory. Such mediation pattern is in accord with previous findings suggesting a limited role for attention in the memory advantage for general negative stimuli (Talmi and McGarry, 2012).

The current dwell-time results are in line with previous findings showing attentional avoidance of disgusting stimuli and disgusted facial expressions (Lazarov et al., 2016, 2021; Armstrong et al., 2019). Disgusted facial expressions were shown to evoke similar neural responses to those evoked by disgusting stimuli (Wicker et al., 2003), it is therefore possible that avoidance of disgusted faces reflect an intentional reaction intended to reduce the unpleasantness of exposure to disgust or a potential disgust experienced by an observed person (Knowles et al., 2019). An alternative explanation for participants’ longer dwelling on neutral relative disgust faces may be associated with the notion that processing of facial expressions is perceptually segmented over time, until a decision is made between several potential expression categories (Jack et al., 2014). Thus, the processing of disgusted expressions might terminate faster than the processing of neutral expressions that do not fall under any expected expression category. This possibility, while cannot be ruled out, appears less likely in the context of the current study because participants were not requested to report or actively process any information regarding the viewed faces during encoding and viewed only two categories of facial expressions (disgust and neutral). Future studies, presenting more diverse sets of facial expressions and involving required decisions regarding expression categories could shed further light on this hypothesis and on the mechanism underlying attentional avoidance of disgusted faces.

The opposite attention and memory patterns noted in the current study are backed-up by the results of the mediation analysis showing independence of memory for disgusted faces from attentional processes at encoding. These results do not seem to support the combined biases hypothesis (Hirsch et al., 2006), proposing that different stages of information processing influence each other and follow a similar pattern. If attention indeed does not contribute much to enhanced memory for disgust faces, what might be the factors underlying this phenomenon? One possibility is that disgust expressions achieve their memory advantage through distinctiveness. Distinctive stimuli in daily life are remembered better than non-distinctive stimuli (McDaniel and Einstein, 1986; Talmi and McGarry, 2012). The effect of distinctiveness on memory has been shown with bizarre images (McDaniel and Einstein, 1986), and general emotional stimuli (Talmi and McGarry, 2012). It is plausible that disgusted expressions are more distinctive than neutral expressions, considering their reduced frequency in daily life. This relative distinctiveness could account for their advantage in recall (but see Barrett et al., 2019). One way of examining the effect of distinctiveness on memory is by presenting separate lists for each type of stimuli at encoding, and using two separate memory tests. Such a design was found to eliminate the memory advantage for general negative stimuli (Talmi and McGarry, 2012). Future studies could use such a design to examine the unique contribution of distinctiveness to better memory for disgust over neutral faces.

Some limitations of the current study are worth noting. First, the use of only one type of negative expression leaves open the possibility that our findings are not generalizable to all negative expressions. There is room for future experiments to examine the same research question using other negative expressions, such as anger and fear, in order to expand the knowledge regarding attention and memory for negative expressions in general. Second, our study does not incorporate an index of strength of the memory traces. This could be measured using a ‘remember/know’ design, enabling to differentiate stimuli that were remembered based on familiarity, or recollection (Gardiner and Java, 1993), or by examining memory for pictorial details. Studies that measured memory for pictorial details of general disgusting images found results in an opposite direction to ours, with poorer memory for details of disgusting images (Fink et al., 2018; Fink-Lamotte et al., 2021). It is possible that although recognition memory for the disgusted expressions in our study was stronger than for neutral expressions, details memory for the former was weaker. A focus on this aspect of memory for disgusted expressions may be a valuable direction for future research. Third, because we used a 2AFC task in the recognition stage we were unable to conduct a signal detection analysis on our memory results, which is a common tool for dissociating between sensitivity and response bias (Stanislaw and Todorov, 1999). Future studies may design a task that fits the requirements for a signal detection analysis in order to acquire a more reliable measure for response bias. Fourth, another direction for future studies could be examining personality traits as possible moderators for the cognitive biases found in our study. Specifically, disgust proneness, i.e., the tendency to experience disgust more frequently, was previously related to attention, interpretation, and memory biases for disgust stimuli (Knowles et al., 2019). Based on these findings, it could be that individuals high or low in disgust proneness would present different pattern of association between attention and memory for disgust faces. Finally, the use of dwell-time in the context of a free viewing task as an indicator for attention allocation leaves room for future studies to explore the attention-memory association for disgust expressions using other established attention tasks (e.g., the modified flanker task, Fenske and Eastwood, 2003), or other eye-tracking measures such as pupil dilation (Hoeks and Levelt, 1993), to provide converting or more nuanced description of this association.

In conclusion, the current study shows that although dwell time on facial expressions has a small effect on memory performance, memory outcomes contradict the attentional patterns in the case of disgusted expressions, with better recognition of disgust over neutral faces occurring in spite of greater attention to neutral faces at encoding. These findings contribute to the understanding of the mechanisms underlying social information-processing, and suggest that perception of social information might be negatively biased, despite attending mostly to neutral cues in the social environment.

## Data availability statement

The datasets presented in this study can be found in online repositories. The names of the repository/repositories and accession number (s) can be found in the article/supplementary material.

## Ethics statement

The studies involving human participants were reviewed and approved by Tel Aviv University ethics committee. The patients/participants provided their written informed consent to participate in this study.

## Author contributions

All authors listed have made a substantial, direct, and intellectual contribution to the work and approved it for publication.

## Funding

This work was partially supported by the Israel Science Foundation (grant number 1811/17).

## Conflict of interest

The authors declare that the research was conducted in the absence of any commercial or financial relationships that could be construed as a potential conflict of interest.

## Publisher’s note

All claims expressed in this article are solely those of the authors and do not necessarily represent those of their affiliated organizations, or those of the publisher, the editors and the reviewers. Any product that may be evaluated in this article, or claim that may be made by its manufacturer, is not guaranteed or endorsed by the publisher.
